# Defining predictive factors for reproductive output in captive common marmosets (*Callithrix jacchus*)

**DOI:** 10.1002/ajp.22926

**Published:** 2018-10-10

**Authors:** Jaco Bakker, Annet L. Louwerse, Edmond J. Remarque, Jan A. M. Langermans

**Affiliations:** ^1^ Animal Science Department Biomedical Primate Research Centre Rijswijk The Netherlands; ^2^ Department of Virology Biomedical Primate Research Centre Rijswijk The Netherlands

**Keywords:** colony management, dam, litter size, marmosets, reproductive output

## Abstract

Common marmosets (*Callithrix jacchus*) demonstrate variations in reproductive output, not only in terms of total reproductive output during a lifetime but also in litter size per parturition. The present study explores factors, such as parents’ litter size, parturition number, maternal body weight at conception and maternal age, which may account for this variation. A retrospective analysis of clinical records of a captive breeding colony was conducted over a 9‐year period yielding reproductive summaries of 26 dams and 22 sires producing a total of 115 litters. Dams born from litters of  ≤2 (*N* = 20) more often produced litters of ≤2, whereas dams born from litters of >2 (*N* = 6) more often produced litters of >2 (*p *< 0.05). The dams’ maternal body weight at the time of conception had also a significant effect on subsequent litter size. In addition, the chance of triplets was higher after the second parturition. Maternal age, interbirth interval, and season of birth had no effect on litter size. Factors relating to the sire had a negligible effect on the size of the litter. Multivariate statistical modeling revealed that the dams’ original litter size, maternal bodyweight at conception and parturition number are determining factors for the number of babies per litter. This study identified factors determining marmoset litter size, some of which (maternal litter size) are novel to this study and were not reported previously. Further exploration of the potential role of maternal litter size as a determinant of the litter sizes produced by marmoset breeders is warranted.

## 1 INTRODUCTION

One of the defining social behaviors of the common marmoset (*Callithrix jacchus*) is their system of cooperative breeding and infant care. The common variation in litter size in captivity is 1–4, with twins and triplets being the most common. However, in the wild, higher proportions of twin litters are observed (Sousa, Silva, & Vidal, 1999). This variation in reproductive output may be related to higher energy availability in captivity resulting in higher body weights. A link between higher maternal body weight and higher ovulation numbers has been previously suggested (Box & Hubrecht, 1987; Rutherford, deMartelly, Layne Colon, Ross, & Tardif, 2014; Tardif & Jaquish, 1997; Tardif et al., 2003).

The most commonly described factors, assumed to affect reproductive output, are maternal body weight, maternal age, parturition number, and increased energy‐protein content of the diet (Ash & Buchanan‐Smith, 2014; Hearn & Burden, 1979; Hearn, Lunn, Burden, & Pilcher, 1975; Jaquish, Cheverud, & Tardif, 1996; Rothe, Darms, & Koenig, 1992; Smucny et al., 2004; Tardif & Jaquish, 1997; Tardif et al., 2003; Tardif, Power, Layne, Smucny, & Ziegler, 2004). However, those studies focused mostly on the relationship between number of ovulations and the effect of intrauterine environments and dams’ longevity, but not on the number of infants produced per litter per parturition.

This study explores factors that may account for the variability in litter size per parturition in captive marmosets and therefore, help in predicting and manipulating litter sizes in captivity. Litter sizes of more than two are not desirable as it is extremely difficult for a female to rear triplets. One infant usually dies or would require hand‐rearing. Therefore, we set out to identify risk factors for litter sizes >2 using a large demographic database of a captive marmoset breeding colony. Defining factors that can predict reproductive output per litter size per parturition (number of infants produced at full term gestation, death and alive) will help to manage captive marmoset breeding programs.

## 2 METHODS

### 2.1 Population description

The demographic data and reproductive information used in this study were obtained from the database records of the marmoset breeding colony at the Biomedical Primate Research Centre (BPRC, Rijswijk, The Netherlands). This marmoset colony was formed in 1975 and consisted initially of marmosets obtained from various accredited suppliers (only captive‐bred marmosets were included). Later, new breeding lines were introduced on several occasions to maintain the outbred character of the colony. The colony continuously includes around 15 breeding groups comprising a total of approximately 150 marmosets, ranging from infants to adults older than 12 years. Marmosets are maintained as monogamous breeding pairs, sharing their enclosure with successive sets of offspring. The offspring remains with their family group for as long as possible, that is, until either the dam or sire or both parents reject them or until they are selected for experimental use (>1.5 years old).

The marmosets are housed in enclosures with a heated indoor compartment and an outdoor compartment; the marmosets are able to move freely between both compartments. Both compartments measure 300 × 200 × 300 cm. The marmosets’ environmental enrichment is optimized by using a complex system of fixed and swinging branches, ropes, nets, and wooden runways. The bedding in the enclosure consists of deep litter (Bakker, Ouwerling, Heidt, Kondova, & Langermans, 2015).

The temperature in the indoor compartment is maintained between 22 and 25 °C with a relative humidity between 50% and 60% and with a 12:12‐h light:dark cycle (lights on, 0700–1900). Lighting in the indoor compartment is provided using full spectrum fluorescent bulbs placed close to the cages in addition to natural light through windows.

The room ventilation rate is around eight air changes per hour. The daily diet consists of commercial primate pellets for New World Monkeys (Sniff, Soest, Germany) offered ad libitum and supplemented with limited amounts of fresh fruit, vegetables, Arabic gum, and homemade porridge. Tap water is provided ad libitum by way of automatic watering nipples.

As part of routine husbandry, weights of all marmosets are obtained, on average, once weekly. Weights are taken by placing a scale in the animal's home cage, using PRT‐training—i.e., marmosets did not have to be handled for weighing. Weights at likely conception dates, approximately 144 days prior to date of birth of the produced litter (Hearn, 1986), were used in the analysis as well as the body weight measurements recorded closest to the date of parturition. All animal procedures, husbandry, and housing were conducted according to BPRC Animal Welfare Body requirements. The research in this study adhered to the American Society of Primatologists' Principles for Ethical Treatment of Non‐Human Primates.

The data used for the current study were obtained between August 2009 and February 2018. The data set consisted a total of 149 births in 32 dams and 25 sires for body weight and inter parturition interval analysis (data set 1). Reproductive output per litter size per parturition was defined as number of infants produced at full term gestation, death and alive. For 34 births in 6 dams, dam's litter size was unknown, leaving a total of 115 births in 26 dams and 22 sires for the analysis of factors influencing litter size (data set 2). For 10 births involving three sires, sire's litter size was not known, therefore analyses involving sire's litter size were performed on a dataset with 105 parturitions in 25 dams (data set 3). Dataset 3 was also used to re‐run models, not involving sire litter size of origin, to allow comparison of AIC values as this requires the same dataset. Age range of the marmoset dams and sires in data set 2 was 2.2–7.9 years and 2.4–7.9 years, respectively.

### 2.2 Statistical analysis

All statistical analyses were performed with the R language and environment for statistical computing version 3.4.3 (R Foundation for Statistical Computing, Vienna, Austria. ISBN 3‐900051‐07‐0, URL http://www.R-project.org). A value of *p* < 0.05 was considered significant.

Multivariate analyses were performed by mixed logistic regression models using the lme4 package, where a litter size >2 was coded 1 and litters ≤2 as 0. Dam was included as a random variable, thereby accounting for pseudoreplication. Several explanatory variables (dams’ litter size of origin, dams’ body weight, >2rd litter for dam, dam age, sires’ litter size of origin) were sequentially added as fixed variables. Collinearity was addressed by calculation of the Variance Inflation Factor (VIF) for each of the explanatory variables in models involving more than one explanatory variable. The final model was selected based on the lowest Aikake Information Criterion. The parameters obtained with the best fitting logistic regression model were subsequently used to construct a triplet “risk” calculator to calculate the probability of a triplet given predictive input parameters.

## 3 RESULTS

**Figure 1:**
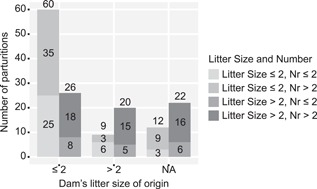
Overview of the dams’ litter size of origin versus produced litter size (n.a. = not available). Most dams were born as part of twin or triplet litters. Dams born from litters of ≤2, produced litters of ≤2 in 70% of cases, whereas dams born from litter size >2 produced litters of >2 in 69% of cases

The produced litter size was significantly affected by the dam's litter size of origin and triplets were less likely in the first two litters (Odds Ratio = 4.95 [95% CI 1.42–17.31], *p* = 0.028) (Figure 1). The third and following parturitions yielded larger litters; after parturition two, the chance of producing triplets was higher (*p* < 0.05) (Odds Ratio = 0.20 [95% CI 0.06–0.71], *p* = 0.012). Parturitions were spread all around the year, no seasonal distribution was observed, and no relation between litter size and moment of the year the parturition took place was observed. No significant effects of dam age on litter size were found. Average litter size was relatively constant during the dams’ lifespan, and even at “older” ages litter sizes did not change. The summary of produced litter size according to dams’ litter size, dams’ body weight and dams’ parturition number >2 is presented as supplementary Figure.

The increase in the dams’ bodyweight just before parturition compared to the body weight at conception was related to the produced litter size. The mean body weight increase of the dam (95% CI) with singletons, twins, or ≥triplets was 83 gr (61 to 105), 88 gr (81 to 95), and 116 gr (109 to 122), respectively.

Inter birth intervals (IBI) did not differ for the produced litter size. The mean IBI (95% CI) with singletons, twins, or ≥triplets was 167 days (145 to 189), 191 days (178 to 204), and 172 days (158 to 186), respectively.

**Figure 2:**
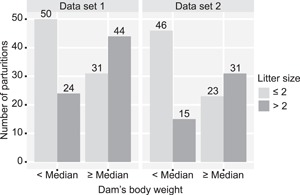
Dams’ body weight at conception versus produced litter size. Dams with body weights below the median at conception produced litters ≤2 in 75% of cases, whereas dams with body weights ≥the median produced litters >2 in 57% of cases

Over 90% of the litter sizes analyzed were multiples (Table 1). Most dams were born as part of twin or triplet litters (Table 2). Dams born from litters of ≤2, produced litters of ≤2 in 70 % of cases, whereas dams born from litter size >2 produced litters of >2 in 69% of cases (Figure 1). Dams born as singleton or twin litters are 2.25 times (1.28–3.94) more likely to produce litters ≤2 (*p *< 0.05), whereas dams born as triplet litters are 2.28 times (1.52–3.41) more likely to produce litters >2 (*p *< 0.05). Dams with body weights below the median at conception produced litters ≤2 in 75% of cases, whereas dams with body weights ≥ the median produced litters >2 in 57% of cases Dams with body weights below the median are 1.78 times (1.26–2.59) more likely to produce litters ≤2 (*p *< 0.05), whereas dams with body weights ≥ the median are 2.33 times (1.42–3.83) more likely to produce litters >2 (*p* < 0.05) (Figure 2).

**Table 1:** Produced litter size divided in singletons, twins, triplets, and quadruplets

	Data set 1				Data Set 2			
Litter size	Dams	Sires	Number of parturitions	Frequency	Dams	Sires	Number of parturitions	Frequency
1	9	7	12	0.0805	8	6	11	0.0957
2	26	21	69	0.4631	20	18	58	0.5043
3	19	19	65	0.4362	14	14	44	0.3826
4	3	3	3	0.0201	2	2	2	0.0174

Overview of the produced litters. Presented in absolute numbers but also in frequency of occurrence. The data set consisted a total of 149 births in 32 dams and 25 sires for body weight and inter parturition interval analysis (data set 1). For 34 births in 6 dams, dam's litter size was unknown, leaving a total of 115 births in 26 dams and 22 sires for the analysis of factors influencing litter size (data set 2). Data show that over 90% of the litter sizes analyzed were multiples.

**Table 2:** Dams’ litter size of origin

Dams’ litter size	Number of dams	Number of parturitions	Frequency
1	2	6	0.0403
2	18	80	0.5369
3	6	29	0.1946
Not available	6	34	0.2282

Overview of the dams litter size of origin with absolute number of parturitions produced and the frequency.

Most sires were born as part of twin and triplet litters (Table 3). Sires born from litters of ≤2 generally produce litters of ≤2. However, this finding was not statistically significant. An interaction between dam and sire litter size of origin was determined but revealed no significant effect.

**Table 3:** Sires’ litter size of origin

	Data set 1			Data set 2		
Sire litter size	Number of sires	Number of parturitions	Frequency	Number of sires	Number of parturitions	Frequency
1	4	15	0.1007	3	11	0.0957
2	8	56	0.3758	8	50	0.4348
3	8	47	0.3154	8	44	0.3826
Not available	5	31	0.2081	3	10	0.0870

Overview of the sires’ litter size of origin. Most sires (>90%) were born as part of twin and triplet litters. Sires born from litters of ≤2 generally produce litters of ≤2.

Logistic regression analysis, accounting for pseudoreplication, was performed with explanatory variables added in a stepwise manner. The best fitting model based on the lowest Aikake Information Criterion revealed that the dams’ litter size of origin, maternal bodyweight and parturition number predicted produced litter size (Table 4). Sires’ litter size of origin showed a small non‐significant effect, nevertheless, same direction as dams. IBI, age and day of the year when parturition took place did not hold predictive value for produced litter sizes.

**Table 4:** Logistic model: triplet produced (0 or 1) explained by: Dam's litter of origin >2 (0 or 1) + dam body weight at conception (in dag) + third or later litter

Variable	Coefficient	Std Err. Coeff	*p*‐value	VIF
Intercept	10.647	3.703	0.004	‐
Dam's litter of origin triplet	2.370	1.078	0.003	1.032
Dam weight (dag)	0.232	0.099	0.002	1.054
Third or later litter	1.599	0.639	0.012	1.086

Multivariate analyses were performed by mixed logistic regression models, where a litter size >2 was coded 1 and litters ≤2 as 0. Dam was included as a random variable, thereby accounting for pseudoreplication. Several explanatory variables (dams’ litter size of origin, dams’ body weight, >2rd litter for dam, dam age, sires’ litter size of origin) were sequentially added as fixed variables. Dam weight was entered as deca‐gram (dag) thus the parameter estimate indicates the change in log Odds for a 10 g weight change.Collinearity was addressed by calculation of the Variance Inflation Factor (VIF) for each of the explanatory variables in models involving more than one explanatory variable. Aikake Information Criterion (AIC) = 119.5282.

To translate from logistic regression estimates to probabilities, a triplet “risk” table (Table 5) was constructed outlining the probability of a triplet given various combinations of the explanatory variables.

**Table 5:** Predicted probability for litters >2, according the explanatory variables

	First or second litter		Third or later litter	
Dams’ weight (g)	Dam originates from litter of ≤2	Dam originates from litter of >2	Dam originates from litter of ≤2	Dam originates from litter of >2
300	0.024	0.209	0.109	0.567
310	0.030	0.250	0.134	0.623
320	0.038	0.296	0.163	0.675
330	0.047	0.346	0.197	0.724
340	0.059	0.400	0.236	0.768
350	0.073	0.457	0.280	0.806
360	0.090	0.515	0.329	0.840
370	0.111	0.572	0.382	0.869
380	0.136	0.628	0.438	0.893
390	0.166	0.680	0.496	0.913
400	0.200	0.728	0.554	0.930
410	0.240	0.772	0.610	0.944
420	0.285	0.810	0.663	0.955
430	0.334	0.843	0.713	0.964
440	0.387	0.871	0.758	0.971
450	0.444	0.895	0.798	0.977
460	0.501	0.915	0.833	0.982
470	0.559	0.931	0.862	0.985
480	0.615	0.945	0.888	0.988
490	0.668	0.956	0.909	0.991
500	0.717	0.964	0.926	0.993

A triplet “risk” table is shown outlining the probability of a triplet given various combinations of the explanatory variables.

## 4 DISCUSSION

The present study shows that the dams’ litter of origin size has predictive value in relation to litter size. Dams born from a twin litter are more likely to give birth to twin litters and dams born from triplet litters are more likely to produce triplets. Therefore, it is tempting to speculate there is a contribution of genetic factors to the produced litter size either as a direct hereditary or epigenetic trait. In addition to the effect of the dams’ litter size, our data show that parturition number and maternal body weight at time of conception significantly influenced the produced litter size. By contrast, the influence of the sire on the litter size is negligible. However, it cannot be excluded that the sire passes on a genetic trait to his daughters without influencing the current litter size.

It has been suggested that marmosets may be able to adjust litter size in pregnancy in response to proximate environmental factors as triplet females were not more likely than twin females to produce triplet litters (Jaquish, Cheverud, et al., 1996; Rutherford et al., 2014; Tardif et al., 2004). However, the data of this long‐term follow‐up study show that the dams litter size of origin had predictive value in produced litter size. It has been suggested that stressors, such as noise disturbance, changes in food availability or maternal body condition, or changes in social setting, may be associated with loss of litter (Tardif & Jaquish, 1997). These losses might be way of controlling reproductive investment and ensure that the number of infants produced reflects the dam's possibility to provide optimal feed and care for them. Rutherford et al. (2014) showed that although there are no differences in the number of offspring between triplet females and twin females, the loss of offspring during pregnancy is significantly higher in triplet females. This manuscript reports a finding that is the opposite of that reported on a different marmoset population (Rutherford et al., 2014), the earlier study reporting that females born into triplet litters were not more likely than twin females to produce triplets. It should be taken into account that the study by Rutherford et al. included 62 dams, with 32 triplet‐born and 30 twin‐born, whereas the present study included 26 dams of which six were known to be of triplet born origin. Despite the low number of dams from triplet litters in this study we did find significant effects, although it can not be fully excluded that this may be an elusive high. The reasons for the different findings between the two studies are not clear. There might be various possible explanations for the observed differences, such as genetic aspects, variations in diets and/or environmental factors (Bakker et al., 2015; Delimitreva et al., 2013). To better explain the observed differences, further research and discussions amongst more centers that breed marmosets would be worthwhile to pursue. Based on the present results, we have decided to limit the inclusion of dams from triplet litters in our breeding colony. It should be noted that only the number of produced offspring was considered in this study and that number of ovulations, prenatal survivorship, and potential in utero reabsorption of fetuses were not included.

Longevity and total reproductive output of the dams were not determined, but it is conceivable that a high reproductive output is at the cost of longevity (Kirkwood, 1977; Westendorp & Kirkwood, 1998). Indirect factors possibly influencing reproductive variation such as group size (i.e., number of potential helpers), and group composition were also excluded from analysis. Where possible, future analysis should include all these parameters.

The influence of the maternal body weight at conception as predictor of litter size may be related to energy availability as marmosets opportunistically adjust their reproductive output in response to changes in diet or energy resources (Jaquish, Tardif, Toal, & Carson, 1996; Tardif & Jaquish, 1997; Tardif et al., 2004). This confirms the link between higher maternal body weight (energy availability) and higher reproductive output (Ash & Buchanan‐Smith, 2014; Box & Hubrecht, 1987; Luke & Keith, 1992; Rutherford et al., 2014; Tardif & Bales, 2004; Tardif & Jaquish, 1997; Tardif et al., 2003; Tardif et al., 2004; Tardif, Ziegler, Power, & Layne, 2005). Most likely, maternal body condition rather than body weight will be a better indicator for litter size. Body condition scoring (BCS) is, however, a subjective semi quantitative method of assessing body fat and muscle by palpation of key anatomic features. The available database is still under development and might be included in future studies.

The effect of maternal age on reproduction in marmosets is not clear, as contradictory data have been published. Twin and triplet litters are common in captivity, with a tendency to increasing numbers of triplet births with time from establishment of a colony (Box & Hubrecht, 1987). This result might have been caused by an abundance of high quality food instead of aging. However, Rothe et al. (1992) describes that age or parity does not affect produced litter size while others even described declining litter size with age (Smucny et al., 2004). In the present study, no age effect on litter size was observed.

The influence of the parturition number on litter size has not been reported before; we observed that triplets are less likely to be produced in the first two litters, but the chance of triplets is increased thereafter.

The absence of seasonal influence on the litter size is in agreement with earlier studies (Box & Hubrecht, 1987; Hearn, 1986). The suggestion of a potential seasonal influence in some wild populations is probably related to the availability of food (Sousa et al., 1999), which is not an issue in captivity.

In general twin and singleton litters do not require food supplementation. However, in captivity, twin and triplet litters are common, which is confirmed in this study. Larger litters have considerably greater perinatal mortality than twin litters, ranging from 30% of infants from triplet litters to 65% from quintuplets (Ash & Buchanan‐Smith, 2014; Jaquish, Gage, & Tardif, 1991). Poor coordination of cooperative parental behavior has been suggested as a cause of this loss (Tardif, Layne, Cancino, & Smucny, 2002), but also infant condition might play a role (Tardif, Layne, & Smucny, 2002). It is clear that, in this species, infants must be able to cling and locomote well, from birth, if they are to survive (Rothe, 1974). Another cause could be due to lack of milk of the dam to feed three infants. Irrespective the cause, one of the three infants of a triplet often loses weight within 2–3 days and dies within a week of birth (Hearn & Burden, 1979; Hearn et al., 1975). Triplets can also be cross fostered if an appropriate dam is available. It must be realized that dams seem to dry off quickly, if the foster dam is more than 3 days out from the last time she nursed, she probably doesn't have any milk left. To improve survival of triplets, infants from triplet litters can be rotationally hand‐reared. In general, this human intervention is necessary to get them through the first 2–3 weeks. Despite the above described human intervention, triplets are still associated with higher infant mortality (Ash & Buchanan‐Smith, 2014; Hearn & Burden, 1979). Hand‐rearing techniques have also serious adverse effects, as normal maternal and family relations are disturbed. The early experiences of marmosets are critical in influencing their development and ability to cope with later events, changes or other stressors (Dettling, Feldon, & Pryce, 2002; Dettling, Schnell, Maier, Feldon, & Pryce, 2007; Pryce, Dettling, Spengler, Schnell, & Feldon, 2004; Pryce et al., 2005). As alternative for the cross fostering and hand‐rearing, one of the infants of a triplet can be euthanized at day 1 to bring total litter size down to two. Besides ethical dilemmas this also has an emotional impact. The human‐animal bond positively impacts quality of life for research animals, but staff caring for the animals often experience euthanasia‐related stress symptoms comparable to those encountered in veterinary clinics and animal shelters. Constant exposure to or participation in euthanasia procedures can cause a psychological state characterized by a strong sense of work dissatisfaction, alienation, or careless and callous handling of animals. The results of this study can potentially be used as an additional measure to the various breeding policies that are currently applied to help preventing production of triplet litters as much as possible.

By defining the factors that predict the produced litter size per parturition, practical aspects of managing marmoset breeding colonies can be enhanced. Maintaining a colony of breeders, with longer healthy life spans and an increased incidence of twin litters could have far reaching implications to improve the quality of life for marmosets in breeding facilities.

## CONFLICT OF INTEREST

The authors of this manuscript have no conflict of interest that would inappropriately bias this research.

## Supporting information

Additional Supporting Information may be found online in the supporting information tab for this article.

Supporting Figure S1.
